# Reduced total arterial compliance in frail older adults is associated with orthostatic blood pressure variability but not baroreflex sensitivity

**DOI:** 10.1016/j.tjfa.2026.100214

**Published:** 2026-10-13

**Authors:** Dihogo Gama de Matos, Felipe J. Aidar, Albena Nunes-Silva, Govind Peringod, Vincenzo Giambusso, Roman Romero-Ortuno, Todd A. Duhamel, Rodrigo Villar

**Affiliations:** a Cardiorespiratory & Physiology of Exercise Research Laboratory, Faculty of Kinesiology and Recreation Management, University of Manitoba, Winnipeg, MB, R3T 2N2, Canada; b Graduate Program in Physical Education and Kinesiology and Physiological Sciences, Federal University of Sergipe, Sao Cristovao, 49100-000, Brazil; c Laboratory of Inflammation and Exercise Immunology, Department of Physical Education, School of Physical Education, Federal University of Ouro Preto, Ouro Preto, MG, 35400-000, Brazil; d Cumming School of Medicine, University of Calgary, AB, T2N 2T8, Canada; e Department of Experimental and Clinical Medicine, University of Florence and Careggi University Hospital, Florence, 50134, Italy; f Discipline of Medical Gerontology, School of Medicine, Trinity College Dublin, Dublin, D02 PN40, Ireland; g Faculty of Kinesiology and Recreation Management, University of Manitoba, Winnipeg, MB, Canada;; h Institute of Cardiovascular Sciences, St. Boniface General Hospital Albrechtsen Research Centre, Winnipeg, MB, Canada.; i Department of Physiology and Pathophysiology, University of Manitoba, Winnipeg, MB, R3E 0J9, Canada; j Centre on Aging, University of Manitoba, Winnipeg, MB, R3T 2N2, Canada.

**Keywords:** Frailty, Arterial compliance, Postural transition, Autonomic regulation

## Abstract

**Background:**

Total arterial compliance (TAC) reflects arterial elasticity and may be associated with baroreflex sensitivity (BRS) and beat-by-beat blood pressure variability (BPV) during postural transitions. Frailty is also associated with impaired autonomic cardiovascular regulation, which may further compromise blood pressure control during orthostatic stress. However, these relationships have not been investigated in frail older adults. We hypothesized that frail participants would exhibit lower TAC than non-frail participants and that reduced TAC would be associated with impaired autonomic regulation.

**Methods:**

Community-dwelling older adults aged 60-79 years were classified as non-frail (*n*=28) or frail (*n*=22) using the Frailty Index. Participants completed sitting-to-standing and lying-to-standing transitions with continuous cardiovascular monitoring. TAC was estimated as the stroke volume-to-pulse pressure ratio, BRS using the sequence method, and BPV using frequency-domain analysis. General linear models compared TAC between groups. Correlation and regression analyses examined associations between TAC, BRS, and BPV, adjusting for frailty status and medication use.

**Results:**

Frail participants exhibited lower TAC than non-frail participants during both conditions (all *p* < 0.05). TAC was not associated with BRS during either orthostatic transition (both *p* > 0.05). In contrast, lower TAC was associated with greater systolic BPV during sitting-to-standing (SBPV-VLF: β = -0.46, *p* = 0.006) and lying-to-standing (SBPV-VLF: β = -0.60, *p* < 0.001; SBPV-LF: β = -0.56, *p* < 0.001; SBPV-HF: β = -0.32, *p* = 0.04).

**Conclusions:**

These findings suggest that impaired arterial compliance contributes to orthostatic autonomic dysfunction in frail older adults primarily through reduced vascular buffering rather than impaired cardiovagal baroreflex function.

## 1 Introduction

Frailty is a multidimensional clinical syndrome characterized by reduced physiological reserve and impaired resilience to internal and external stressors, resulting in increased vulnerability to adverse health outcomes [1,2]. Among the physiological systems affected by frailty, the cardiovascular system undergoes structural and functional alterations that reduce the ability to maintain arterial pressure during daily activities, particularly during orthostatic challenges [2,3]. Consequently, frail older adults are at increased risk of orthostatic intolerance, falls, hospitalization, and loss of independence [4,5]. Orthostatic intolerance may result not only from increased beat-by-beat BPV [6] but also from a pronounced BP reduction after standing, as observed in orthostatic hypotension [7]. Therefore, both the magnitude of the initial BP decline and subsequent BP stability may influence orthostatic tolerance.

One of the hallmark features of cardiovascular aging is the progressive reduction in arterial elasticity. Total arterial compliance (TAC), an index reflecting the ability of the arteries to expand and recoil with each cardiac cycle, progressively declines with age because of arterial stiffening and vascular remodeling [8]. Emerging evidence suggests that frailty may further impair vascular compliance beyond the effects of chronological aging [9,10], potentially limiting the cardiovascular system's capacity to buffer acute changes in blood pressure (BP) during postural transitions [2,11].

Maintenance of BP during standing depends on the integrated interaction between the arterial system’s mechanical properties and autonomic regulation [12]. Following a postural transition, arterial baroreceptors detect the reduction in arterial pressure and initiate rapid autonomic adjustments that increase heart rate, modify vascular resistance, and restore BP [12]. Beat-by-beat BP variability (BPV), particularly its low-frequency component, which reflects peripheral vasomotor activity and is influenced by sympathetic vascular modulation, provides complementary information on the mechanisms underlying BP regulation [13,14]. Because TAC influences the transmission and buffering of pulsatile pressure throughout the arteries [15], alterations in arterial compliance may contribute to changes in both baroreflex sensitivity (BRS) and BPV during orthostatic stress.

Although previous studies have independently demonstrated impairments in vascular function [16], autonomic regulation [17], and orthostatic BP control in frailty [2], the relationship between TAC and autonomic regulation during active standing remains poorly understood. Furthermore, it remains unclear whether reduced arterial compliance is associated with BRS and beat-to-beat BPV during orthostatic transitions after accounting for frailty status. Because cardiovascular responses are influenced by the magnitude of gravitational stress, examining transitions from different starting postures may provide complementary information about orthostatic regulation. Sitting-to-standing represents a lower gravitational load and a common daily movement, whereas lying-to-standing imposes a higher gravitational load and elicits larger immediate BP reductions and cardiovascular adjustments [18].

Therefore, the primary aim of this study was to compare TAC between frail and non-frail older adults during sitting-to-standing and lying-to-standing. The secondary aim was to investigate the associations between TAC, BRS, and beat-by-beat BPV and to determine whether TAC predicts autonomic regulation after adjusting for frailty status and medication. We hypothesized that frail participants would exhibit lower TAC than non-frail participants and that reduced TAC would be associated with altered BRS and BPV during orthostatic transitions.

## 2 Methods

The University Research Ethics Board granted ethical approval for the study under the protocol number HE2022-0058.

### 2.1 Participants

Participants were recruited using a convenience sampling approach. Community-dwelling older adults representing a range of frailty levels were identified through recruitment sources, including Dr. Todd Duhamel's Women's Advanced Risk-Assessment in Manitoba (WARM Hearts) study database (for female participants) [19], Centre on Aging participant registry (mainly females), and the Men's Shed organization (for male participants). Individuals interested in participating contacted the research team by telephone or email to receive additional information and undergo eligibility screening.

The study included community-dwelling older adults (60-79 years) who completed orthostatic stress assessments consisting of sit-to-standing and lying-to-standing transitions. Eligible participants were required to be within the specified age range, assigned female or male at birth, and able to stand independently without assistance. Individuals were excluded if they had a history of cardiovascular disease (including ischemic heart disease, stroke, coronary artery bypass graft surgery, or congestive heart failure), psychiatric disorders, or severe cognitive impairment. Information on medication use was collected during the study visit and included as a covariate.

Before the laboratory assessment, participants were instructed to avoid caffeine and alcohol consumption and to refrain from strenuous physical activity for at least 48 hours. Adherence to these pre-assessment instructions and confirmation of the study eligibility were verified upon arrival at the laboratory. Participants then received a standardized explanation of the study procedures, and written informed consent was obtained before any experimental testing was conducted.

Each participant completed two active standing protocols in a randomized, counterbalanced order: (1) sit-to-standing protocol consisting of 5 minutes of seated rest followed by 7 minutes of standing, and (2) lying-to-standing protocol comprising 10 minutes of supine rest followed by 7 minutes of standing. The baseline periods were selected to ensure cardiovascular stabilization before the postural transition [11], where only the last 3 minutes of baseline in each condition were used for analysis.

A total of 124 individuals were initially contacted; 60 met the eligibility criteria and were enrolled. Beat-to-beat cardiovascular recordings from 11 participants during the sit-to-stand protocol and 10 during the lie-to-stand protocol were excluded because excessive motion artifacts, signal loss, or recording errors compromised data quality. Consequently, the final analysis included 49 participants for the sit-to-stand transition (28 non-frail and 21 frail) and 50 participants for the lie-to-stand transition (28 non-frail and 22 frail) (Fig. 1).

**Fig. 1:**
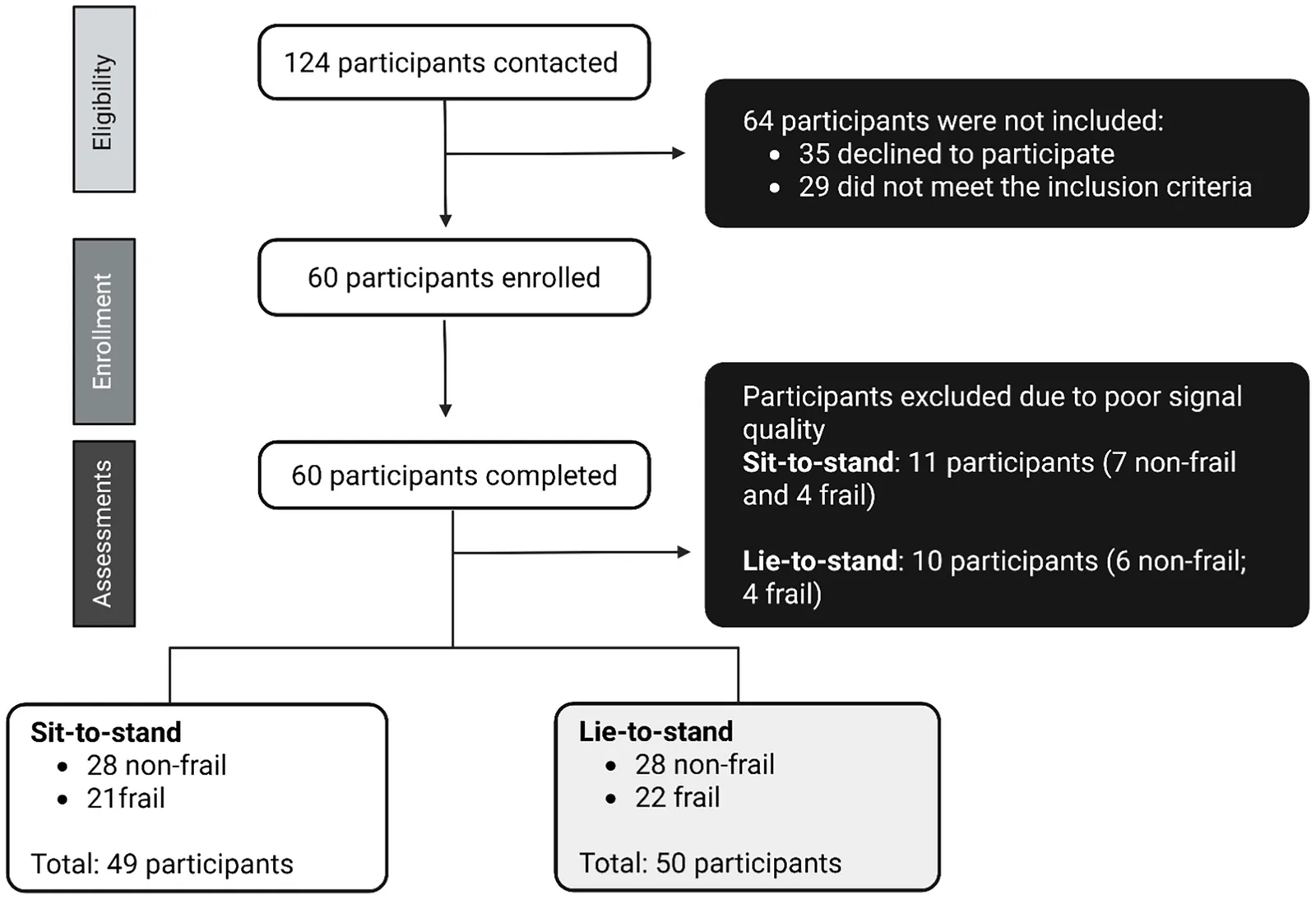
Recruitment flow chart of the participants.

### 2.2 Demographic, anthropometric measurements, and frailty assessment

Demographic and anthropometric characteristics, including sex assigned at birth, age, height, body mass, and body mass index (BMI), were recorded for all participants. Frailty status was determined using the Frailty Index (FI) as a continuous measure, but was also used as a categorical variable to classify participants as non-frail (FI ≤ 0.08), pre-frail (FI >0.08 to <0.25), or frail (FI ≥ 0.25), according to established recommendations [20]. The mean FI was 0.04 ± 0.03 in the non-frail group, 0.12 ± 0.03 in the pre-frail group, and 0.32 ± 0.04 in the frail group. Because of the relatively small number of participants classified as frail (*n* = 8) and pre-frail (*n* = 14), the frailty group was combined into a single group for all analyses to ensure adequate statistical power.

### 2.3 Assessment of cardiovascular responses

Heart rate and BP were continuously monitored throughout both orthostatic protocols using non-invasive instrumentation. Heart rate (HR) was derived from R-R intervals obtained from a three-lead electrocardiogram sampled at 1 kHz. Systolic blood pressure (SBP), diastolic blood pressure (DBP), and mean arterial pressure (MAP) were measured continuously using finger photoplethysmography (Finometer^TM^ Pro, Finapres Medical Systems BV, Amsterdam, The Netherlands). Approximately five minutes before each protocol, return-to-flow calibration was performed to adjust finger arterial pressure to brachial arterial pressure, followed by manual calibration against an automated upper-arm BP measurement (ARSIMAI, BSX516, Munster, Germany). A height correction system was used to reference finger pressure to heart level throughout the assessment. The calibrated SBP and DBP values were subsequently used to calculate pulse pressure. Stroke volume (SV) was estimated from the arterial pressure waveform using the Modelflow algorithm [21] (Finapres Medical Systems, Arnhem, The Netherlands).

### 2.4 Total arterial compliance (TAC) measurements

Total arterial compliance (TAC) was estimated noninvasively using the stroke volume-to-pulse pressure ratio, a simplified index derived from the two-element Windkessel model [22,23]. Although no accepted noninvasive gold standard exists for assessing TAC, the SV/PP approach has been extensively validated and has been shown to provide a reliable estimate of global arterial compliance, with good agreement with other commonly used Windkessel-based methods [22,24,25]. Moreover, comparative studies have demonstrated that the SV/PP method is among the most robust approaches for evaluating arterial compliance in clinical and research settings [25,26]. SV was derived from the Finometer using the Modelflow method [27], while SBP and DBP were recorded beat-to-beat. Pulse pressure (PP) was calculated as the difference between beat-to-beat SBP and DBP. TAC was calculated as the beat-to-beat ratio of SV/PP, with beat-to-beat TAC averaged over the first minute following the postural transition.

### 2.5 Baroreflex sensitivity

Cardiovagal baroreflex sensitivity (BRS) was assessed using the spontaneous sequence method [28]. This technique identifies sequences of three or more consecutive cardiac cycles in which progressive increases or decreases in SBP are followed by corresponding lengthening or shortening of the R-R interval. Minimum beat-to-beat changes of 1 mmHg for SBP and 6 ms for the R-R interval were required for a sequence to be accepted. Then, the slope of the linear relationship between SBP and the R-R interval (ms.mmHg^-1^) was calculated [28,29]. Only sequences with a correlation coefficient (*r)* > 0.85 were included in the analysis [28,29]. Cardiovagal BRS was calculated from all accepted sequences identified during the 7-minute standing period following each orthostatic transition, and the mean slope was then used in the analyses.

### 2.6 Beat-by-beat blood pressure variability (BPV)

Beat-by-beat BPV was assessed from continuous SBP and DBP recordings using frequency-domain spectral analysis from the first minute of the standing phase following each orthostatic transition. Beat-to-beat SBP and DBP values were resampled at 10 Hz, and autoregressive spectral analysis was performed separately for SBP and DBP to quantify BPV within the predefined frequency bands [13]. The spectral components analyzed included the very-low-frequency band (VLF = <0.04 Hz), which primarily reflects slow vascular regulatory mechanisms, including myogenic vascular activity, endothelial function, and humoral influences such as the renin-angiotensin system [30,31]. The low-frequency band (LF = 0.04-0.15 Hz) serves as an index of sympathetic vascular modulation [32]. The high-frequency (HF = 0.15-0.40 Hz) is largely influenced by respiratory-related fluctuations in cardiac output [30].

### 2.7 Sample size

A power analysis was conducted using G*Power 3.1 (Heinrich Heine University, Düsseldorf, Germany) [33] for an F test using ANCOVA (fixed effects, main effects, and interactions). The analysis was based on the primary comparison of TAC between non-frail and frail participants, with medication included as a covariate. The analysis assumed an effect size of *f* = 0.52 (partial η^2^ = 0.21), α = 0.05, two groups, and one covariate. The estimated total sample size required to achieve 80% statistical power (1 - β) was 32 participants.

### 2.8 Statistical analysis

Statistical analyses were performed using Jamovi (The Jamovi Project, Sydney, Australia - version 2.6.44), with significance set at *p* < 0.05. Descriptive data are presented as mean ± standard deviation (SD) and range (minimum-maximum). Normality was assessed using the Shapiro-Wilk test. The primary analysis evaluated differences in TAC between frail and non-frail participants during the sitting-to-standing and lying-to-standing transitions using a general linear model (GLM). Frailty status (non-frail and frail) was included as the between-subject factor, condition (sitting baseline, sitting-to-standing, supine baseline, and lying-to-standing) as the within-subject factor, and medication as a covariate. Main effects and frailty-by-condition interactions were examined. Although the frailty-by-condition interaction was not significant, Bonferroni-adjusted post hoc comparisons were used to characterize differences between frailty groups within each condition. These comparisons were retained because the primary objective was to examine whether TAC differed between non-frail and frail participants during each orthostatic protocol. Partial eta-squared (η²p) was calculated to estimate effect sizes. Effect size was classified as low (≤ 0.05), medium (0.06-0.25), high (0.26-0.50), and very high (> 0.50) [34]. Secondary analyses investigated the associations between TAC, BRS, and BPV. Because several variables did not satisfy the assumption of normality, associations were evaluated separately within frail and non-frail participants using Spearman’s rank correlation coefficient (ρ). Correlation magnitudes were interpreted using the absolute value of Spearman’s correlation coefficient (ρ) as small (0.10-0.29), medium (0.30-0.49), and large (≥0.50) [34].

Multiple linear regression analyses were subsequently performed to explore whether TAC was associated with BRS and BPV after adjusting for frailty status and medication. TAC and medication were entered as covariates, whereas frailty status was entered as a categorical factor. Medication was recorded for all participants and classified according to antihypertensive drug class [35]. Because several participants were receiving combination therapy and the number of participants within each medication category was insufficient for meaningful subgroup analyses (see Table 1), medication was included as a single covariate in the general linear models and multiple linear regression analyses. Standardized regression coefficients (β), coefficients of determination (*R*^2^), and corresponding *p*-values are reported. Model assumptions were evaluated by inspection of residual plots and the Shapiro-Wilk test of residuals.

**Table 1:** Sample characteristics of participants by Frailty status.

Variables	Non-frail (*n* = 28)		Frail (*n* = 22)		
**Anthropometric**	Mean ± SD	Min; Max	Mean ± SD	Min; Max	*p*
Age	70.3 ± 3.8	63; 77	70.8 ± 4.1	63; 78	0.6
Height (m)	1.6 ± 0.1	1.5; 1.8	1.6 ± 0.0	1.5; 1.8	0.9
Body mass (kg)	73.6 ± 15.6	46.6; 110	73.7 ± 15.4	42.5; 109	0.9
BMI	26.4 ± 4.6	17.1; 36.6	28.5 ± 6.7	17.9; 48.4	0.1
**Hemodynamic**					
Systolic blood pressure	127 ± 12	108; 154	132 ± 13	110; 150	0.1
Diastolic blood pressure	77 ± 7.3	56; 92	77 ± 8.8	60; 92	0.1
Heart rate	66 ± 7.8	52; 84	76 ± 8.7	64; 92	<0.001*
**Medication**	***n* (%)**	-	***n* (%)**	-	**-**
Any antihypertensive medication	14 (50)	-	15 (68)	-	**-**
Calcium channel blockers	9 (32)	-	10 (46)	-	**-**
Beta-blockers	5 (18)	-	6 (27)	-	**-**
Diuretics	3 (11)	-	6 (27)	-	**-**
ACE inhibitor	9 (32)	-	0 (0)	-	**-**
Angiotensin receptor blocker	0 (0)	-	5 (23)	-	**-**

Data are presented as mean and SD with Minimum (Min) and Maximum (Max); BMI: body mass index. Anthropometric and hemodynamic *p*-values derived from an independent t-test. Medication use is presented descriptively and was included as a covariate in subsequent analyses. *Statistically significant differences between the non-frail and the frail group.

## 3 Results

Table 1 summarizes the demographic, anthropometric, and baseline hemodynamic characteristics of the participants according to frailty status. Age, height, body mass, body mass index, and resting SBP and DBP were similar between groups (all *p* > 0.05). Compared with non-frail participants, the frail group exhibited a higher resting HR (all *p* < 0.01). Overall, 14 non-frail (50%) and 15 frail participants (68%) were receiving at least one antihypertensive medication. Owing to the small number of participants within individual medication classes and the frequent use of combination therapy, class-specific analyses of medication were not performed.

Table 2 presents the GLM analysis of TAC during the sitting-to-standing and lying-to-standing transitions. A significant main effect of frailty status was observed (*p* < 0.001), indicating consistently higher TAC in non-frail than frail participants. Non-frail participants exhibited significantly higher TAC than frail participants at the sitting baseline (*p* = 0.009), sitting-to-standing (*p* < 0.001), and lying-to-standing (*p* = 0.02). No statistically significant differences were observed at supine baseline between groups (*p* = 0.06). Medication had a significant effect on TAC (*p* = 0.01). Neither the frailty-by-condition interaction nor the main effect of condition was statistically significant.

**Table 2:** General linear model analysis of total arterial compliance during sitting-to-standing and lying-to-standing transitions.

Variable	Condition	Group	Baseline	Standing
TAC (mL/mmHg)	Sitting-to-standing	Non-frail	1.20 ± 0.26^†^ (0.65 - 1.71)	1.29 ± 0.3^†^ (0.73 - 1.97)
		Frail	0.97 ± 0.14 (0.60 - 1.18)	1.00 ± 0.13 (0.65 - 1.22)
			
	Lying-to-standing	Non-frail	1.15 ± 0.26 (0.78 - 1.81)	1.24 ± 0.31^†^ (0.64 - 1.86)
		Frail	0.90 ± 0.16 (0.55 - 1.30)	1.00 ± 0.20 (0.57 - 1.52)
		Interaction (*p*, η²p)	0.8 (0.004)	
		Main condition effect (*p*, η²p)	0.1 (0.002)	
		Main group effect (*p*, η²p)	<0.001* (0.21)	
		Medication	0.01* (0.03)	

Data are presented as mean ± SD with minimum and maximum values (Min-Max). Group differences were analyzed using a general linear model (GLM) adjusted for medication. Main effects are reported as *p*-values with partial eta squared (η²p). TAC - total arterial compliance. ^†^Significantly different from the frail group within the same condition based on Bonferroni-adjusted post hoc comparisons (*p* < 0.05). *Statistically significant *p* < 0.05.

Table 3 summarizes the Spearman correlation analyses between TAC, BRS, and BPV during the sitting-to-standing and lying-to-standing transitions. During the sitting-to-standing transition, no statistically significant associations were observed between TAC and BRS or BPV in non-frail participants. In frail participants, TAC was not associated with BRS or diastolic BPV (all *p* > 0.05) but showed large negative correlations with systolic BPV VLF (*p* = 0.01), LF (*p* = 0.009), and HF (*p* = 0.002). During the lying-to-standing transition, non-frail participants showed no association between TAC and BRS or diastolic BPV (all *p* > 0.05). However, TAC demonstrated large negative correlations with systolic BPV VLF (*p* = 0.001) and LF (*p* = 0.002), and a medium negative correlation with systolic BPV HF (*p* = 0.04). In frail participants, TAC was not associated with BRS. However, TAC demonstrated large negative correlations with systolic BPV VLF (*p* = 0.02) and LF (*p* = 0.01), medium negative correlations with systolic BPV HF (*p* = 0.03) and diastolic BPV LF (*p* = 0.03), and a large negative correlation with diastolic BPV VLF (*p* = 0.02). No significant association was observed between TAC and diastolic BPV-HF. Supplementary Figure 1 shows representative beat-by-beat ΔSBP tracings from a non-frail and a frail participant during the lying-to-standing transition.

**Table 3:** Spearman correlations between total arterial compliance, baroreflex sensitivity, and blood pressure variability during the sitting-to-standing transition.

**Variable**		**Non-frail**				**Frail**	
**Sitting-to-standing**	Spearman's ρ	95% CI		*p*-value	Spearman's ρ	95% CI	*p*-value
BRS (ms/mmHg)	-0.06	-0.436, 0.349		0.7	-0.27	-0.623, 0.142	0.2
SBPV - VLF (ms^2^)	-0.22	-0.600, 0.217		0.3	-0.53	-0.795, 0.020	0.01*
SBPV - LF (ms^2^)	-0.13	-0.577, 0.351		0.5	-0.55	-0.822, -0.126	0.009**
SBPV - HF (ms^2^)	-0.18	-0.577, 0.299		0.4	-0.63	-0.848, -0.220	0.002**
DBPV - VLF (ms^2^)	-0.23	-0.615, 0.212		0.2	-0.38	-0.759, 0.140	0.08
DBPV - LF (ms^2^)	-0.18	-0.602, 0.271		0.4	-0.40	-0.757, 0.094	0.07
DBPV - HF (ms^2^)	-0.14	0-.575, 0.346		0.5	-0.27	-0.671, 0.214	0.2
**Lying-to-standing**							
BRS (ms/mmHg)	-0.01	-0.401, 0.365		0.9	0.23	-0.297, 0.605	0.3
SBPV - VLF (ms^2^)	-0.57	-0.773, -0.270		0.001**	-0.65	-0.826, -0.132	0.02*
SBPV - LF (ms^2^)	-0.55	-0.753, -0.238		0.002**	-0.53	-0.793, -0.113	0.01*
SBPV - HF (ms^2^)	-0.38	-0.641, -0.012		0.04*	-0.47	-0.723, 0.065	0.03*
DBPV - VLF (ms^2^)	-0.3	-0.610, -0.006		0.07	-0.51	-0.652, 0.324	0.02*
DBPV - LF (ms^2^)	-0.01	-0.389, 0.353		0.9	-0.47	-0.610, 0.421	0.03*
DBPV - HF (ms^2^)	-0.02	-0.388, 0.316		0.9	-0.43	-0.611, 0.387	0.06

Values are presented as Spearman's rank correlation coefficients (ρ), 95% confidence intervals (95% CI), and corresponding *p*-values. Correlations were performed separately for non-frail and frail participants. BRS - baroreflex sensitivity; SBPV - systolic blood pressure variability; DBPV - diastolic blood pressure variability; VLF – very-low-frequency; LF – low-frequency; HF – high-frequency. *Statistically significant *p* < .05, ^⁎⁎^*p* < .01.

Table 4 presents the multiple linear regression analyses examining associations of TAC with BRS and BPV after adjusting for frailty status and medication. During the sitting-to-standing transition, TAC was inversely associated with systolic BPV-VLF (*p* = 0.006), diastolic BPV-VLF (*p* = 0.01), diastolic BPV-LF (*p* = 0.01), and diastolic BPV-HF (*p* = 0.03), whereas no associations were observed with BRS, systolic BPV-LF, or systolic BPV-HF (all *p* > 0.05). Frailty status remained independently associated with BRS (*p* < 0.001). Medication was associated only with systolic BPV-VLF (*p* = 0.02). During the lying-to-standing transition, TAC was associated with systolic BPV-VLF, systolic BPV-LF, systolic BPV-HF (all *p* < 0.04), and diastolic BPV-VLF (*p* = 0.004). No significant associations were identified with BRS, diastolic BPV-LF, or diastolic BPV-HF (all p > 0.05). Frailty status remained an independent predictor of BRS (*p* = 0.002) and systolic BPV-VLF (*p* = 0.009). Medication was not associated with any outcome during the lying-to-standing transition (all *p* > 0.05).

**Table 4:** Multiple linear regression analyses examining associations between total arterial compliance, baroreflex sensitivity, and blood pressure variability during sitting-to-standing and lying-to-standing transitions.

		**TAC**			**Frailty**		**Model**
**Sitting-to-standing**	β	95% CI	*p*-value	β	95% CI	*p*-value	*R*^2^
BRS (ms/mmHg)	0.05	-0.680, 0.98	0.70	**-1.09**	-1.329, -0.38	<0.001*	0.36
SBPV - VLF (ms^2^)	**-0.46**	-2260.6, -412	0.006**	-0.29	-761.6, 290	0.30	0.25
SBPV - LF (ms^2^)	-0.33	-195, 1.60	0.05	-0.11	-65, 46.86	0.70	0.13
SBPV - HF (ms^2^)	-0.24	-46.58, 8.26	0.10	-0.34	-22.93, 8.29	0.30	0.06
DBPV - VLF (ms^2^)	**-0.4**	-850.7, -106	0.01*	-0.30	-303.3, 120	0.40	0.16
DBPV - LF (ms^2^)	**-0.43**	-74.53, -9.79	0.01*	-0.29	-26.26, 10.59	0.30	0.19
DBPV - HF (ms^2^)	**-0.36**	-18.45, -0.61	0.03*	-0.41	-8.07, 2.07	0.20	0.14
**Lying-to-Standing**	β		*p*-value	β		*p*-value	*R*^2^
BRS (ms/mmHg)	0.02	-0.716, 0.85	0.80	**-0.95**	-1.21, -0.28	0.002**	0.23
SBPV - VLF (ms^2^)	**-0.60**	-2411, -940	<0.001**	**-0.73**	-1027, -156	0.009**	0.36
SBPV - LF (ms^2^)	**-0.56**	-169.7, -55.01	<0.001**	-0.47	-61.8, 6.07	0.10	0.26
SBPV - HF (ms^2^)	**-0.32**	-37.42, -0.13	0.04*	-0.39	-17.71, 4.35	0.20	0.09
DBPV - VLF (ms^2^)	**-0.45**	-762, -155.2	0.004**	-0.50	-328, 31	0.10	0.18
DBPV - LF (ms^2^)	-0.29	-54.97, 2.58	0.07	-0.24	-23.53, 10.52	0.40	0.08
DBPV - HF (ms^2^)	-0.15	-10.87, 4.03	0.30	-0.22	-5.87, 2.95	0.50	0.02

Multiple linear regression models included total arterial compliance (TAC), frailty status, and medication as independent variables. Values are presented as standardized regression coefficients (β), corresponding 95% confidence intervals for the unstandardized regression coefficients (B), and *p*-values for TAC and frailty status. Medication coefficients are not shown because medication was included as an adjustment variable in all models; medication was significantly associated only with systolic BPV-VLF during the sitting-to-standing transition. Variance Inflation Factor (VIF) values for all predictors were < 2, indicating no evidence of multicollinearity. BRS - baroreflex sensitivity; SBPV - systolic blood pressure variability; DBPV - diastolic blood pressure variability; VLF – very-low-frequency; LF – low-frequency; HF – high-frequency. *Statistically significant *p* < 0.05, ^⁎⁎^*p* < .01.

## 4 Discussion

The present study is the first to investigate TAC in frail older adults during active orthostatic transitions and to examine its relationship with BRS and beat-by-beat BPV. The principal findings were threefold. First, frail participants exhibited significantly lower TAC than non-frail participants, indicating impaired arterial compliance in frailty. Second, TAC was not associated with cardiovagal BRS during either orthostatic transition, suggesting that reduced arterial compliance does not necessarily translate into impaired vagal baroreflex function. Third, TAC demonstrated consistent associations with beat-by-beat BPV. These findings suggest that reduced arterial compliance contributes primarily to altered vascular pressure regulation during orthostatic stress rather than to impaired cardiovagal baroreflex control.

## 5 Frailty is associated with reduced arterial compliance

Reduced arterial compliance is a well-established consequence of vascular aging and reflects progressive structural and functional alterations within the arterial wall, including elastin fragmentation, collagen accumulation, endothelial dysfunction, and vascular remodeling [[36], [37], [38]]. Frailty appears to accelerate these age-related vascular changes through chronic inflammation, oxidative stress, reduced physical activity, and impaired physiological reserve [[39], [40], [41]]. Consequently, arteries become less capable of expanding during systole and recoiling during diastole, reducing their ability to buffer pulsatile blood flow [42].

Our findings extend this concept by demonstrating that lower TAC is consistently present in frail older adults during both baseline and active standing conditions. Importantly, these differences remained evident regardless of orthostatic condition, indicating that reduced arterial compliance is an intrinsic characteristic of frailty rather than merely a transient response to standing. This observation supports the hypothesis that vascular dysfunction represents an important component of the frailty phenotype and may contribute to the impaired cardiovascular adaptation commonly observed during orthostatic stress.

## 6 Lack of association between TAC and baroreflex sensitivity

TAC was not associated with BRS after accounting for frailty status and medication. Although arterial stiffening has often been linked to reduced baroreflex function [43], our findings suggest that vascular mechanical properties and neural baroreflex regulation may represent distinct physiological mechanisms during active standing. BRS primarily reflects the efficiency of neural cardiovascular reflexes, including baroreceptor activation, afferent signaling, central autonomic integration, and efferent vagal responses [44,45]. In contrast, TAC represents the passive mechanical properties of the arterial system [26]. Although arterial compliance and BRS both contribute to short-term BP regulation during orthostatic stress, arterial compliance primarily reflects the mechanical buffering properties of the arterial system, whereas spontaneous BRS reflects neural cardiovascular reflex function, highlighting their distinct vascular and neural roles [29,38,42]. The absence of an association between TAC and BRS suggests that vascular mechanical dysfunction may influence orthostatic blood pressure regulation without proportionally affecting cardiovagal baroreflex gain.

## 7 Reduced arterial compliance is associated with greater beat-to-beat blood pressure variability

A major finding of the present study was the consistent inverse association between TAC and beat-to-beat BPV. Individuals with lower arterial compliance demonstrated greater systolic BPV during both orthostatic transitions, with the strongest associations observed during the lying-to-standing protocol. These findings suggest that reduced arterial elasticity limits the artery's capacity to buffer rapid pressure fluctuations immediately following postural change.

According to the Windkessel principle, compliant arteries temporarily store part of the stroke volume during systole and release it during diastole, thereby dampening pressure oscillations and maintaining a more stable arterial pressure [46]. As arterial compliance decreases, this buffering capacity is progressively lost, allowing greater transmission of pulsatile energy through the artery and increasing beat-to-beat blood pressure fluctuations [46]. During active standing, when venous return transiently decreases and arterial pressure must be rapidly stabilized, this reduced buffering capacity may amplify pressure oscillations and contribute to orthostatic instability [47].

These findings are consistent with previous observations in hypertensive populations demonstrating relationships between TAC and BPV [26]. Our findings extend these observations by demonstrating that this relationship is also present in frail older adults during active orthostatic stress, a physiological condition that imposes substantially greater demands on short-term cardiovascular regulation than resting assessments. Also, our findings suggest that reduced arterial compliance may contribute to impaired orthostatic blood pressure regulation primarily through diminished vascular buffering capacity rather than alterations in cardiovagal control of the heart. However, sympathetic baroreflex-mediated vasoconstriction may also contribute to beat-by-beat blood pressure regulation during orthostatic stress. Because muscle sympathetic nerve activity and regional sympathetic vasoconstrictor responses were not assessed, their contribution to the observed associations between TAC and BPV cannot be determined [48,49].

## 8 Clinical implications

The present findings have important clinical implications. Orthostatic intolerance, falls, and functional decline are common complications of frailty [50], yet the vascular mechanisms contributing to these outcomes remain incompletely understood. Our results suggest that TAC may represent a useful physiological marker of vascular dysfunction associated with impaired blood pressure regulation during orthostatic stress. Because TAC can be estimated non-invasively using beat-to-beat hemodynamic recordings [26] it may provide a practical approach for identifying older adults with reduced cardiovascular reserve who may benefit from interventions targeting vascular health, including structured exercise and lifestyle modification.

## 9 Limitations

The cross-sectional design precludes conclusions regarding causal relationships between reduced arterial compliance and altered orthostatic blood pressure regulation. Although lower TAC was associated with greater beat-to-beat BPV, the temporal direction of this relationship cannot be established, and both variables may reflect common frailty-related vascular changes [51,52]. Also, TAC was estimated using the SV/PP ratio, an accepted non-invasive surrogate of arterial compliance. Because PP is derived from SBP minus DBP, TAC and BPV are not entirely mathematically independent. Consequently, part of the observed association between TAC and BPV may reflect mathematical coupling rather than a purely physiological relationship. Nevertheless, TAC also incorporates SV, and BPV reflects the temporal variability of blood pressure rather than PP itself, indicating that the observed associations are unlikely to be explained solely by shared mathematical components. Additionally, only cardiovagal BRS was assessed. Direct measures of sympathetic baroreflex function, muscle sympathetic nerve activity, and sympathetic vasoconstrictor responses were not obtained. Therefore, the contribution of sympathetic vascular control to the associations between TAC and BPV cannot be determined. Future studies should incorporate direct measurements of sympathetic baroreflex function, such as muscle sympathetic nerve activity and regional vascular responses, to clarify how sympathetic vasoconstrictor control interacts with arterial compliance to influence BPV in frail older adults. Also, future studies should confirm these findings using independent measures of arterial stiffness or compliance, such as carotid-femoral pulse wave velocity or other validated indices of arterial stiffness [23,27,53].

Although medication was included as a covariate in all statistical models, antihypertensive medications were analyzed as a single variable because of the limited sample size and the frequent use of combination therapy. Different antihypertensive drug classes have distinct effects on vascular function, arterial compliance, and autonomic regulation [35]. Therefore, some residual confounding related to medication class or specific pharmacological effects cannot be excluded. Future studies with larger samples should evaluate the influence of individual antihypertensive classes on orthostatic cardiovascular regulation.

Lastly, although the sample size was adequate for the primary comparison of TAC between frailty groups, the correlation and regression analyses were exploratory and involved multiple associations across autonomic outcomes. Consequently, these findings should be interpreted as hypothesis-generating and require confirmation in larger prospective studies.

## 10 Conclusion

Frailty is associated with reduced total arterial compliance during active orthostatic transitions. Lower arterial compliance is associated with greater beat-to-beat BPV but not with spontaneous BRS, suggesting that vascular mechanical dysfunction contributes to impaired orthostatic blood pressure regulation through mechanisms distinct from neural baroreflex control. These findings identify total arterial compliance as a promising marker of vascular function in frail older adults and provide new insight into the vascular mechanisms underlying orthostatic autonomic regulation in frailty.

## Funding

This research was partially supported in part by the Manitoba Medical Service Foundation (MMSF #2021-11), Centre on Aging Research Fellowship (University of Manitoba), University of Manitoba Retirees Association Scholarship, Center on Aging Betty Havens Memorial Graduate Fellowship, and Jack MacDonell Scholarship for Research in Aging.

## Ethical Standards

The University Research Ethics Board granted ethical approval for the study under the protocol number HE2022-0058.

## Declaration of generative AI use

AI was not used in the development of this project.

## CRediT authorship contribution statement

**Dihogo Gama de Matos:** Project administration, Writing – review & editing, Writing – original draft, Formal analysis, Data curation, Visualization. **Felipe J. Aidar:** Writing – review & editing, Data curation. **Albena Nunes-Silva:** Writing – review & editing, Validation, Investigation. **Govind Peringod:** Writing – review & editing, Software, Data curation. **Vincenzo Giambusso:** Writing – review & editing, Software, Data curation. **Roman Romero-Ortuno:** Writing – review & editing, Validation. **Todd A. Duhamel:** Project administration, Writing – review & editing, Supervision, Formal analysis, Conceptualization. **Rodrigo Villar:** Writing – review & editing, Visualization, Validation, Supervision, Project administration, Methodology, Investigation, Funding acquisition, Conceptualization.

## Declaration of competing interest

The authors declare that they have no known competing financial interests or personal relationships that could have appeared to influence the work reported in this paper.
