# Supplementary material for: Reduced total arterial compliance in frail older adults is associated with orthostatic blood pressure variability but not baroreflex sensitivity
Source: J Frailty Aging. 2026 Oct 13;15(6):100214. doi: 10.1016/j.tjfa.2026.100214 (PMC13685980; doi:10.1016/j.tjfa.2026.100214)
Supplement: Supplementary file 1 [file mmc1.docx]

**Supplementary Figure S1**. Representative systolic blood pressure responses during the lying-to-standing transition in non-frail and frail older adults. Changes in systolic blood pressure (ΔSBP) are presented relative to baseline for one representative non-frail (black line) and one representative frail (red line). The vertical dotted line indicates the transition to standing at 0 seconds. The 120-second period preceding standing represents the supine baseline, followed by 420 seconds of standing. These individual traces are provided for visual illustration and should not be interpreted as group-average responses.
